# Spatiotemporal Dynamics and Fitness Analysis of Global Oil Market: Based on Complex Network

**DOI:** 10.1371/journal.pone.0162362

**Published:** 2016-10-05

**Authors:** Ruijin Du, Gaogao Dong, Lixin Tian, Minggang Wang, Guochang Fang, Shuai Shao

**Affiliations:** 1 Nonlinear Science Research Center, Jiangsu University, Zhenjiang, Jiangsu, China; 2 School of Mathematics Sciences, Nanjing Normal University, Nanjing, Jiangsu, China; 3 School of Economics, Nanjing University of Finance and Economics, Nanjing, Jiangsu 210023, China; 4 Center for Polymer Studies and Department of Physics, Boston University, Boston, Massachusetts, United States of America; Universidad Rey Juan Carlos, SPAIN

## Abstract

We study the overall topological structure properties of global oil trade network, such as degree, strength, cumulative distribution, information entropy and weight clustering. The structural evolution of the network is investigated as well. We find the global oil import and export networks do not show typical scale-free distribution, but display disassortative property. Furthermore, based on the monthly data of oil import values during 2005.01–2014.12, by applying random matrix theory, we investigate the complex spatiotemporal dynamic from the country level and fitness evolution of the global oil market from a demand-side analysis. Abundant information about global oil market can be obtained from deviating eigenvalues. The result shows that the oil market has experienced five different periods, which is consistent with the evolution of country clusters. Moreover, we find the changing trend of *fitness* function agrees with that of gross domestic product (GDP), and suggest that the fitness evolution of oil market can be predicted by forecasting GDP values. To conclude, some suggestions are provided according to the results.

## 1. Introduction

Oil shortage makes the oil flow between different countries an important component of world’s oil trading system. Oil supply risk, which is contained in global oil trading system, has become a central issue of national energy security [1]. It is thus crucial to understand the evolution of world oil trade structure and flow.

In fact, oil trade flows embody the relationship among different countries, which can form a network where the nodes are the countries, and the edges are the oil trade relationships. Therefore, complex network theory can be a helpful tool when analyzing the trading patterns generated by the oil flows. This has been shown in Refs. [2–7]. Ji et al. identified the global oil trade patterns using complex network theory, discovering that the global oil export core network displays the typical feature of complex network, that is scale-free distribution [2]. An et al. constructed a directed network model of international oil trade to study the relationship between countries with common trade parters [3]. In Ref. [4], a weighted complex network model was designed for examing the dynamics of the co-movement between crude oil features and spot prices. Gao et al. studied the features and evolution of international fossil energy trade relationships by using a weighted multilayer network method [5]. Zhong et al. studied the evolution of communities of the world oil trade network by setting up un-weighted and weighted oil trade network models using data from 2002–2011, and analyzed their evolutionary features and stabilities over the time [6]. Zhang et al. expanded their descriptions of trade relations to include competition among oil importers [7]. Ref. [8] built a network model using ecological network analysis to holistically evaluate the security of the crude oil supply in China. A detailed understanding of oil trading-based network is meaningful for governments because they are eager to increase their understanding of global oil trade in order to avoid oil supply risk. Ensuring oil supply safety is a main task for countries all over the world. It is generally known that oil-exporting countries have played an essential role in international oil-trading network. Sudden change in oil-exporting countries will cause fluctuation of oil supply. It has a strong influence on the competition and cooperation relations between oil-exporting countries. Much work has been done on the supply side [9–13].

On the other hand, facing fierce competition in complex international oil market, importers cannot afford to choose exporters and import volume only based on their own demands. Incomplete information and market uncertainties make it difficult for an importer to make an optimal rational decision on its own. Because oil-importing countries are embedded in the oil trading network, their choices are heavily influenced by other importers’ behavior and decisions. When existing studies explore oil trading system on demand side, the correlation and interaction of oil importers has long been neglected [14–16]. However, the intense correlation of imports of oil-dependent countries will lead to blind competition, which will cause the oil importers be at a disadvantage and face threats to supply security in the long term [17, 18]. Thus, research of correlation among oil-dependent countries has an important practical significance. A novel approach to understand such correlations is random matrix theory (RMT), which was introduced in mathematical statistics by Wishart in 1928 [19]. RMT has been used to deal with the correlation matrix structure of financial markets [20–23]. When the correlations among the constituents of a market become stronger, the ripple effect increases and the market risk increases. There is evidence that measures based on eigenvalues and eigenvectors of the correlation matrix can help to characterize market integration, quantify systemic risks measured by absorption ratio, and construct profitable investment portfolios [24–29]. Recent work has started to apply RMT to complex network. Feher et al. constructed a correlation network by choosing a correlation threshold [30]. Kumar and Deo investigated the network properties of world indices during the financial crisis of 2008 by applying RMT and complex network methods [31]. In this work, we construct a set of correlation matrices from oil importers. The study of RMT here has the potential to bridge the micro-level (oil importing country) behavior and the macro-level (oil market) space-time structure, and to reveal the systemic risk and spatiotemporal dynamics of global oil market.

In this paper, Section 2 introduces the theoretical background of complex network theory and random matrix theory. The main empirical analyses for oil-trading patterns and spatiotemporal dynamics of oil market are presented in Section 3. Finally, conclusions are drawn in Section 4.

## 2. Materials and Methods

### 2.1 Complex network theory

#### 2.1.1 Oil trade network modeling

Complex network theory is a powerful system-oriented modeling technique used to examine the structure and flow of oil in oil-trading system. We build a time sequence of weighted, directed global oil trade networks. In the networks, the countries are taken as the nodes and trade relationships as the edges. The exports and imports represent the energy flow out of and into a country respectively, and trading volume is taken as the weight of the edge.

#### 2.1.2 The topological properties of oil trade network

According as the global oil trade includes import and export flows, the network can be split into two subnetworks. One is oil import network, in which the in-degree and in-strength of nodes are only considered. The other one is oil export network, in which the out-degree and out-strength of nodes are only considered. Here the in-degree *k*^*in*^ of a country is used to measure the number of countries, which export oil to that country. The out-degree of a country *k*^*out*^ counts the number of countries, which import oil from that country. The degree of a country *k* is used to measure the number of countries that have direct oil-trading relationships with that country. The strength *s* of a country is an extended definition of degree that measures the total weight of its connected links. The in-strength *s*^*in*^ of a country means the total imports, and the out-strength *s*^*out*^ is the total exports. Obviously, we have *k* = *k*^*in*^ + *k*^*out*^, *s* = *s*^*in*^ + *s*^*out*^.

The overall features of global oil trade network can reflect the oil trade patterns directly at global level. Thus, three specific indicators are applied for the analysis of overall structural characteristics of global oil trade network: cumulative distribution, information entropy and weighted clustering coefficient.

(1) Cumulative distribution

The cumulative distribution of a network can completely describe the distributing characteristic of node degree and strength. The cumulative degree distribution is defined as

$$CP_{k}=\sum_{K\geq k}P\left(K\right),$$ (1)

where *P*(*K*) is the probability of a randomly chosen node in the network with degree *K*. In the same way, we obtain the cumulative strength distribution *CP*_*s*_ = ∑_*S* ≥ *s*_ *P*(*S*), where *P*(*S*) means the fraction of nodes with strength *S* in the network.

(2) Information entropy

The network information entropy reflects the correlation between a node and its neighbor nodes, and characterizes the scale-free character of the network. The information entropy of the network is defined as follows [32]:

$$E=-\sum_{i}I_{i}\log I_{i},$$ (2)

where *I*_*i*_ = *k*_*i*_/∑_*i*_ *k*_*i*_ is the importance of node *i*. Consider a network is completely homogeneous, *E*_*max*_ = log *N* can be obtained, where *N* is the total number of nodes. If all the nodes in a network are connected to one central node, then we get the minimum information entropy $E_{min}=\frac{1}{2}\log4(N-1)$. The standard information entropy is defined as $E_{s}=\frac{E-E_{min}}{E_{max}-E_{min}}$.

(3) Weighted clustering coefficient

In the global oil trade network, the clustering coefficient of country *i* is used to measure the probability that any two countries that have trade relations with *i* also have trading relationship with each other. The weighted clustering coefficient for weighted networks is defined as follows [32]:

$$C_{i}=\frac{\Sigma_{j,k}{\left({\hat{w}}_{ij}{\hat{w}}_{ik}{\hat{w}}_{jk}\right)}^{\frac{1}{3}}}{k_{i}\left(k_{i}-1\right)},$$ (3)

$$\left\langle C\right\rangle =\frac{\Sigma_{i=1}^{N}C_{i}}{N},$$ (4)

$$C\left(k\right)=\frac{\Sigma_{k_{i}=k}C_{i}}{NP(k)},$$ (5)

here the edge weights *w*_*ij*_ are normalized by the maximum weight in the network, ${\hat{w}}_{ij}=w_{ij}/max\left(w\right)$, *N* represents the total number of nodes, < *C* > denotes the average clustering coefficient of the whole network and *C*(*k*) represents the average clustering coefficient over the nodes with degree *k*.

### 2.2 Random matrix theory

Random matrix theory, by comparing statistical characteristics of random multidimensional time series, can reflect the degree that real data deviates from the random, and reveal the overall correlation behavior of real data. RMT provides a useful metric by which we can quantify the type and degree of randomness present in a system. Perhaps most usefully, the statistical properties of RMT help to determine how a system’s large-scale behavior will react to perturbations and failures in the network.

In this subsection, we briefly introduce the basic theory of RMT. We denote *d*_*i*_(*t*) as the monthly oil imports value of country *i*(*i* = 1, 2, …, *m*) at time *t*. For each moving window [*t* − *T* + 1, *t*] at time *t* with size *T*, we compute the correlation matrix *C*(*t*), whose element *C*_*ij*_ is the Pearson correlation coefficient between the import value time series of countries *i* and *j*,

$$C_{ij}\left(t\right)=\frac{1}{\sigma_{i}\sigma_{j}}\sum_{k=t-T+1}^{t}\left[d_{i}\left(k\right)-\mu_{i}\right]\left[d_{j}\left(k\right)-\mu_{j}\right],$$ (6)

where *μ*_*i*_ and *μ*_*j*_ denote the sample means, *σ*_*i*_ and *σ*_*j*_ are the standard deviations of the two countries *i* and *j* respectively.

For the correlation matrix *C*(*t*) at time *t*, we get *m* eigenvalues *λ*_*i*_. Sort the eigenvalues *λ*_*i*_ in descending order and calculate the corresponding eigenvectors *u*_*i*_(*t*) = [*u*_*i*,1_(*t*), …, *u*_*i*, *m*_(*t*)]^*T*^. Then we can measure oil market risk by using the following absorption ratio [24–27]

$$E_{n}\left(t\right)=\sum_{i=1}^{n}\frac{\lambda_{i}}{N},$$ (7)

which is a better approach because perfectly integrated market can exhibit weak correlation. The bigger the absorption ratio is, the greater the market risk is. For each eigenvalue *λ*_*i*_, the corresponding eigenportfolio is [24]

$$D_{i}\left(t^{\prime }\right)=k_{i}\left(t\right)D\left(t^{\prime }\right),$$ (8)

where *t*′ = *t* − *T* + 1, …*t*, *d*(*t*′) = [*d*_1_(*t*′), …, *d*_*m*_(*t*′)]^*T*^. To evaluate the collective market effect embedded in *λ*_*i*_, we investigate the following linear regressive model between *D*_*i*_(*t*′) and *D*(*t*′) [24],

$$D_{i}\left(t^{\prime }\right)=k_{i}\left(t\right)D\left(t^{\prime }\right)+\varepsilon \left(t^{\prime }\right),$$ (9)

where *D*(*t*′) is the mean value of *d*_*i*_(*t*′)(*i* = 1, 2, …, *m*), *D*_*i*_(*t*′) and *D*(*t*′) are normalized respectively to zero mean and unit variance, and *k*_*i*_(*t*) is the correlation coefficient between *D*_*i*_(*t*′) and *D*(*t*′) in time *t*′. If *k*_*i*_ differs significantly from 0, the eigenvalue *λ*_*i*_ is assumed to contain a market effect, since the corresponding eigenportfolio is correlated with the entire market. This implies that if *k*_*i*_ is larger, the market effect is stronger.

## 3. Empirical research

### 3.1 Data

The monthly data on global oil trade is downloaded from UN Comtrade database (http://www.trademap.orgCountry_SelProductCountry_TS.aspx), which contains oil export and import flows among 173 countries in the world. The used code is HS 270900: crude petroleum oils. The yearly data retrieved from U.S. Energy Information Administration is the consumption of petroleum (http://www.eia.govcfappsipdbprojectiedindex3.cfm?tid=5&pid=57&aid=1&cid=regions&syid=2009&eyid=2013&unit=TBPD#). The GDP data can be retrieved from International Monetary Fund (http://www.imf.orgexternaldata.htm). We selected the annual data of all the countries from 2001 to 2014. The following “oil”, unless otherwise indicated, means “crude petroleum oil (HS 270900)”.

### 3.2 Topological structure of oil trade network

**Fig 1:**
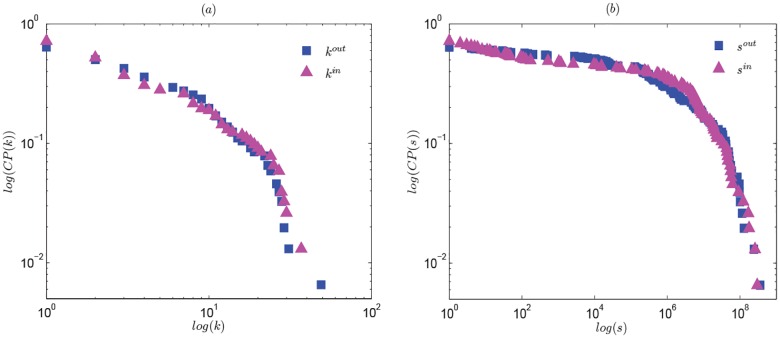
The relationship of cumulative degree distribution and degree, cumulative strength distribution and strength in log-log coordinate of 2013.

Before studying the topological structure of oil trade network, the degree distribution must first be analyzed. If the degree distribution of a network follows a power law, it is a scale-free network, in which a few nodes have major connections, most nodes have a few. The plot of cumulative degree distribution versus degree in log-log coordinate is a straight line. Based on Eq (1), we calculate the cumulative degree and strength distributions of global oil trade network. The results are demonstrated in Fig 1.

Fig 1 shows that both the global oil import and export networks are not typically scale-free. This conclusion is different from that of Ref. [2]. The main reason is that oil importers tend to trade and cooperate with neighboring countries to improve their oil supply security. The selection mechanism of SF network cannot be fully realized.

**Fig 2:**
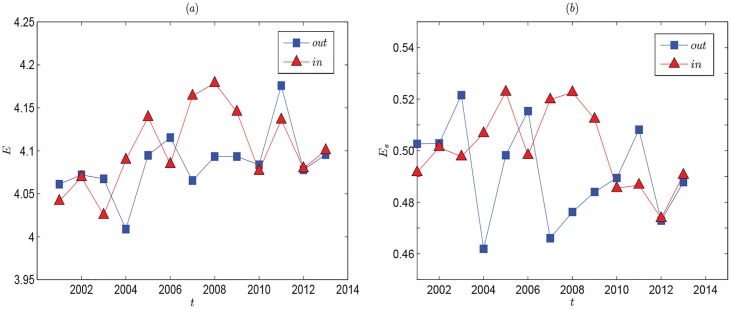
Evolution of information entropy and standard information entropy of global oil trade network. The overall trend is increasing gradually and slowly. It means the global oil trade network tends to be homogeneous slightly.

Another index analyzing the scale-free property quantitatively is information entropy. Serrano et al. [33] found that the world trade network displayed SF degree distribution by using the data of year 2000. The information entropy of import and export world trade network in 2000 were 4.51 and 4.30, and the standard information entropy were 0.707 and 0.616 separately [34]. From Fig 2, the information entropy presents a tendency of increasing. However, the entropy values are far below that of world trade network. This shows that the global oil import and export networks present more obvious heterogeneous characteristics.

**Fig 3:**
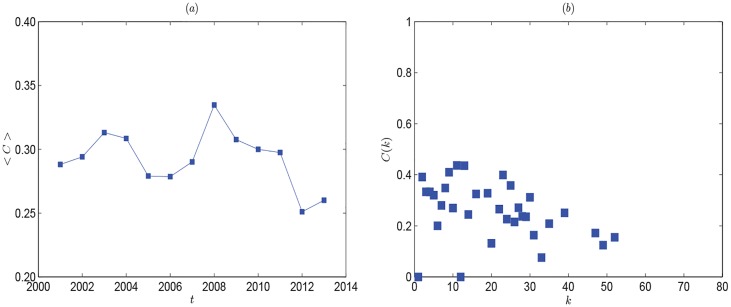
(Left panel:) Evolution of average weighted clustering coefficients for the global oil trade network. The peak point shows the effect of transition brought by the financial crisis of 2008. (Right panel:) The relations of *C*(*k*) and *k* in 2013.

As the generalization of clustering coefficient, the weighted clustering coefficient can reflect the effects of a market crash which is hardly observed with other clustering characteristics studied [32]. The left panel of Fig 3 shows the evolution line of the average weighted clustering coefficient 〈*C*〉 increases dramatically by 2008. It illustrates the connectivity of countries trading with the same one increases. The global oil trade network tends to orderly coordination. But affected by the financial crisis, 〈*C*〉 decreases after 2008. From the right panel of Fig 3, *C*(*k*) decreases as *k* increases. Countries with lower degrees are always with small economies. They always trade with other neighboring countries, so their trading partners have a higher chance of trading with each other. Countries with higher degrees are just the opposite.

Figs 2 and 3 show the evolution of information entropy and weighted clustering coefficient, which are based on a set of network models for each period in the whole sample time interval. Based on the above, we have analyzed the overall characteristics of oil import and export networks. We find the probability densities of degree and strength are not typically scale-free in recent ten years, but the oil import and export networks still show heterogeneous feature, which means the local small countries still prefer to trade with regional core countries. Just because more than one oil importer must inevitably import oil from the same oil exporter, which will force the importers to compete with each other for oil resources. This always lead to excessive competition among oil importers, and cause oil market turmoil. Furthermore, by analyzing the evolution of the number of countries with annual oil trading volume in excess of 1 million tons (see Table 1), we find the variance that is used to measure how far a set of numbers are spread out from their mean, is 16.859 for the sequence of the numbers of importing countries, which is much bigger than 3.4231 for exporting countries. Maybe it’s because the supply of oil is always determined by natural endowment, while the demand of oil is not. Thus, the demand side analysis of global oil trade is necessary. In the following part, by applying RMT analysis, we investigate the correlation of import values from demand side, which can help us better understand the spatiotemporal dynamics and evaluate the fitness of global oil market, can also help avoid negative competition within oil importers and improve the oil supply security.

**Table 1:** Evolution of the number of countries with annual oil trading volume in excess of 1 million tons during 2001–2013.

	01	02	03	04	05	06	07	08	09	10	11	12	13	*Variance*
*No. of oil importers*	56	60	63	62	64	58	62	60	57	56	56	53	50	16.8590
*No. of oil exporters*	51	49	50	52	56	52	53	52	52	50	49	51	51	3.4231

### 3.3 RMT analysis from demand side

First, we need to find major oil-dependent countries by ranking oil import dependency of the world countries. Oil import dependency is often used to measure oil supply risk of a country and is computed as

$$D_{i}=\frac{NI_{i}}{T_{i}},$$ (10)

where *NI*_*i*_ denotes net oil imports, *NI*_*i*_ = ∑_*j* → *i*_ *Q*_*ji*_ − ∑_*i* → *k*_ *Q*_*ik*_, *Q*_*ji*_ indicates oil imports from country *i* to country *j*, what we use *T*_*i*_ here is the petroleum consumption of country *i*.

Based on Eq (10), we get an oil import dependency ranking of 173 countries. We find that oil importers during 2011–2013 are almost the same. There are 53 oil-dependent countries, where 51%, 25%, 11%, 5%, 4% and 4% are from Europe, Asia, Africa, South America, North America and Oceania respectively. Most of the crude oil importers are in Europe and Asia. From the above 53 countries, we choose every month the oil imports value of every country from January 2005 to December 2014 as the sample data. Due to limit and imperfect of data information, finally 38 countries are chosen for the study. 22 countries are from Europe, including Italy(ITA), Germany(GER), Netherlands(NED), Sweden(SWE), Finland(FIN), Lithuania(LTU), Spain(ESP), Poland(POL), Belgium(BEL), Austria(AUT), Greece(GRE), Croatia(CRO), UK, Hungary(HUN), France(FRA), Portugal(POR), Switzerland(CH), Romania(ROM), the Czech Republic(CZE), Ireland(IRL), Bulgaria(BUL) and Slovakia(SVK); 8 countries are from Asia, including India(IND), Singapore (SIN), Thailand(TH), Uzbekistan(UZB), China(CHN), South Korea(KOR), Philippines(PHI) and Japan(JPN); 3 countries are from Africa, including South Africa(SA), Zambia(ZAM) and Morocco(MAR); 2 countries are from Oceania, including Australia(AUS) and New Zealand(NZL); 2 countries are from South America, including Peru(PER) and Chile(CHI); and the United States(USA) is from North America.

The sample data we choose is from January 2005 to December 2014. The sliding-window method means that slice the imports time series of the 38 countries with partially superposed windows. For each window, the correlation matrix is calculated, and the response time is determined. Here, we slice the time series into “24-month periods” lagged by 1 month (see Table 2). The first time window is from January 2005 to December 2006, and the last one is from January 2013 to December 2014. Therefore, there exist 97 time windows, giving us 97 38-order correlation matrices for investigation.

**Table 2:** Principle of the sliding-time windows method. The time series of oil imports are sliced with 23 superposed windows.

*Date*	ITA	SIN	NED	⋯	POL	JPN	GER
*200501*	1992646	1763810	2175419	⋯	341878	5024213	2901491
*200502*	1957991	1582007	1999188	⋯	451657	4919724	2700006
*⋯*	⋯	⋯	⋯	⋯	⋯	⋯	⋯
*200612*	3316372	1527487	2970810	⋯	4717589	8522144	4324030
*200701*	3141860	1795462	2978347	⋯	718893	7381021	4031213
*⋯*	⋯	⋯	⋯	⋯	⋯	⋯	⋯
*201301*	4141017	3905967	6575416	⋯	1485052	12317277	6723621
*⋯*	⋯	⋯	⋯	⋯	⋯	⋯	⋯
*201412*	2197529	1736853	3843483	⋯	981762	9249757	4075812

(1) Correlation coefficient

**Fig 4:**
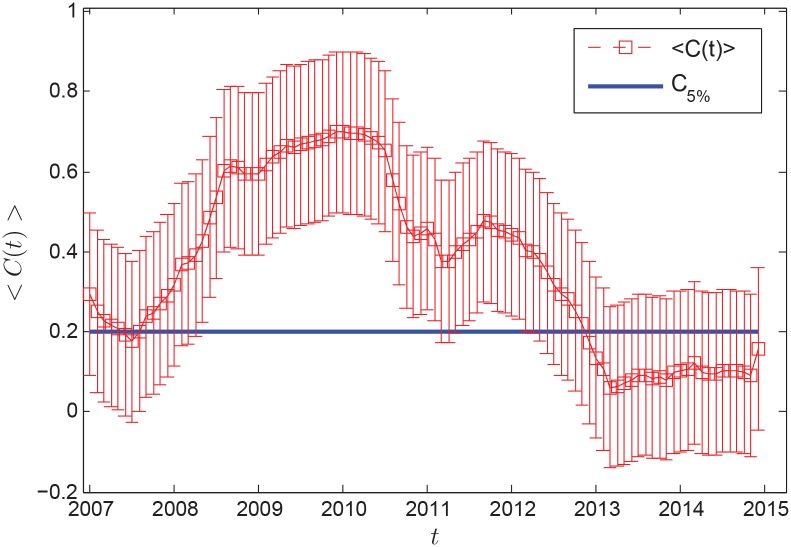
Evolution of average correlation coefficient. The horizontal blue line represents the critical value at significance level 5% of the correlation coefficient at each time *t*.

Fig 4 shows the average correlation coefficient 〈*C*(*t*)〉 of Eq (6) calculated from December 2006 to December 2014. We find 〈*C*(*t*)〉 decreased from the end of 2006 to the middle of 2007. It showed that the oil importers were weakly correlated. The global oil market was relatively stable. However, 〈*C*(*t*)〉increased dramatically from the middle of 2007 to the second half of 2008. It indicated that oil-dependent countries were strongly correlated to each other, and the risk of oil market increased sharply. By the end of 2010, 〈*C*(*t*)〉 fluctuated greatly, which showed oil market was unstable. From the end of 2010 to early 2013, 〈*C*(*t*)〉 decreased obviously, oil importers became weakly correlated. In the two years that followed, 〈*C*(*t*)〉 remained small, the global oil market developed steadily.

(2) Eigenvalues and absorption ratio

**Fig 5:**
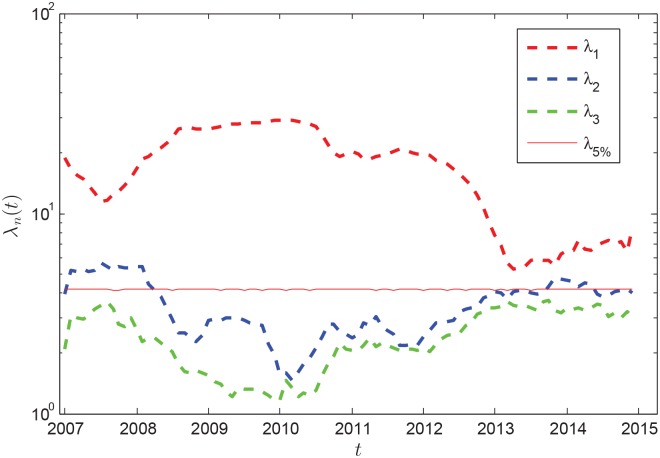
Evolution of the three largest eigenvalues *λ*_*i*_(*i* = 1, 2, 3) of 〈*C*(*t*)〉. The horizontal red line shows the critical values *λ*_5%_ of eigenvalues at the significance level of 5% at each time *t*.

We calculate a correlation matrix *C*_*Rnd*_ from the randomized time series, and compute the corresponding 38 eigenvalues. Repeating this procedure 1000 times, we get a total of 38000 eigenvalues based on which the probability density of eigenvalues *f*_*Rnd*_(*λ*) is obtained. Fig 5 shows that the largest eigenvalue *λ*_1_ of *C*(*t*) has trended upward sharply during the middle of 2007 to late of 2010, coinciding with the bursting of the world-wide financial crisis. For the first largest eigenvalue, we find *λ*_1_ > *λ*_5%_ for all *C*(*t*) matrices, *λ*_2_ is larger that *λ*_5%_ for part of the *C*(*t*) matrices, and the third largest eigenvalue *λ*_3_ falls below *λ*_5%_ for all *C*(*t*) matrices. The eigenvalues *λ*_1_ and *λ*_2_ should thus contain information about nontrivial spatiotemporal properties of global oil market.

**Fig 6:**
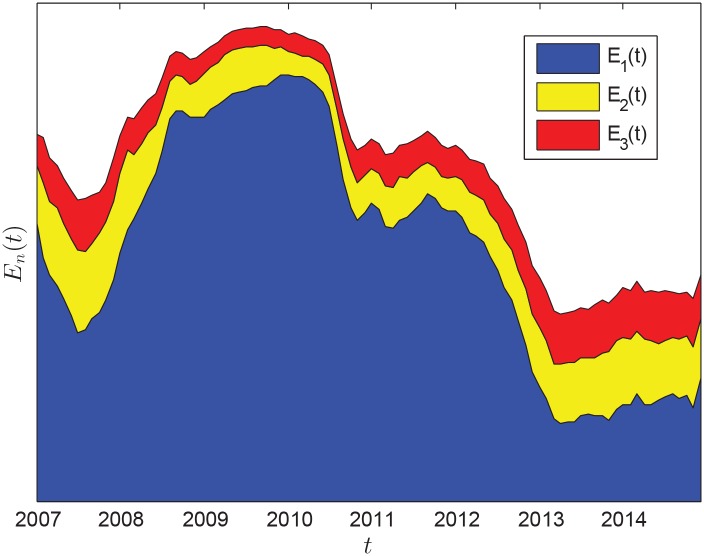
Evolution of absorption ratio *E*_*i*_(*t*) for *i* = 1, 2, 3.

Based on Eq (8), Fig 6 shows the evolution of absorption ratio, the trend of which is very close to that of average correlation coefficient shown in Fig 4. This means a high importing correlation between the major importers indicates a high risk of oil market. At the beginning, the risk of global oil market was low. From the middle of 2007 to the middle of 2008, systemic risk growed linearly. By the end of 2010, or early 2011, systemic risk shows an abrupt downward trend. The risk of oil market declined substantially by early 2013, and then rose slowly and steadily after that.

(3) Period shifts and evolution of countries clusters

**Fig 7:**
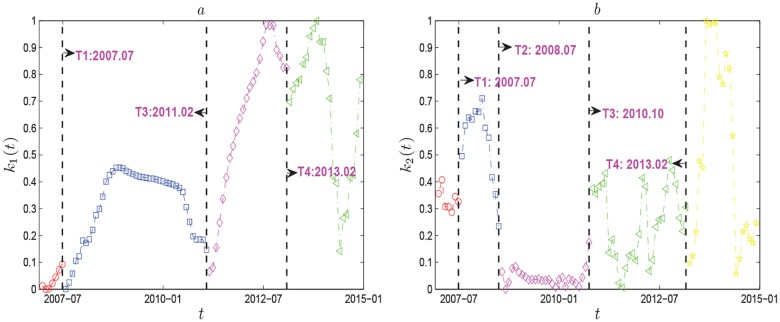
Market effect hidden in the largest eigenvalues. Each symbol shows the evolution of the correlation coefficient *k*_*n*_(*t*) between *D*_*n*_ and *D* in each moving window. The vertical lines indicate the period-shift points.

For each eigenvalue *λ*_*i*_(*i* = 1, 2) and the corresponding eigenvector *u*_*i*_ = [*u*_*i*,1_, …, *u*_*i*,38_]^*T*^, we construct the eigenportfolio based on Eqs (8) and (10) to investigate the linear regressive model. From Fig 7(a) we find the correlation coefficient *k*_1_ between *D*(*t*′) and *D*_1_(*t*′) drops from 0.0931 (July 2007) to 0 (August 2007), then fluctuates largely, and decreases from 0.1469 (February 2011) to 0.0655 (March 2011), then grows sharply, and drops from 0.824 (February 2013) to 0.6972 (March 2013). In this way we can approximately identify four periods, which are surprisingly identical to those we find for *λ*_1_ in Fig 5. For the second largest eigenvalue *λ*_2_, Fig 7(b) shows four period shifts: July 2007-August 2007, July 2008-August 2008, October 2010-November 2010 and March 2013-April 2013, which are close to the regime shifts found by *λ*_1_. Thus from the above, we can identify four period shift points, *T*_1_: 2007.07, *T*_2_: 2008.07, *T*_3_: 2010.10 − 2011.02, *T*_4_: 2013.02, and obtain five eigenvalue periods: *P*_1_ = [2006.12, *T*_1_], *P*_2_ = [*T*_1_, *T*_2_], *P*_3_ = [*T*_2_, *T*_3_], *P*_4_ = [*T*_3_, *T*_4_], *P*_5_ = [*T*_4_, 2014.12]. *k*_1_ in periods *P*_3_, *P*_4_ and *P*_5_ is obviously different from 0, and *k*_2_ in periods *P*_1_, *P*_2_ and *P*_5_ differs significantly from 0. In period *P*_2_, the subprime mortgage loan has developed into a comprehensive global financial crisis, brought great impact on crude oil market, and increased the risk of oil supply. In period *P*_3_, this once-in-a-century financial crisis brought losses to the development of the global economy, and caused global oil market to increase to the highest point. At the end of 2009, the world economy recovered from the crisis gradually, the risk of oil market began a slow decline. In period *P*_4_, oil market affected by multiple factors, like volatile situation in the Middle East, unsolved European debt crisis and the quantitative easing policy, appeared turbulence patterns. By 2012, the new quantitative easing program implemented by major world economies, and the solution to Eurozone debt crisis reached by European summits, which led to a strong recovery of world economy and a sharp decrease of oil market risk. In period *P*_5_, the growth of the world economy slowed down, and oil market risk increased slowly. In addition, we find besides the largest eigenvalue, the second one can also reflect the behavior of global oil market. This is substantially different from the results obtained for stock market. Because for stock market behavior, only the largest eigenvalue reflects the behavior, the other eigenvalues do not [24].

**Fig 8:**
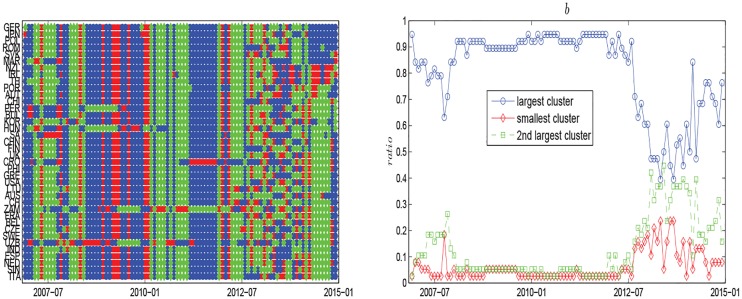
(a) Clustering evolution of oil importers. (b) Evolution of cluster ratios.

**Fig 9:**
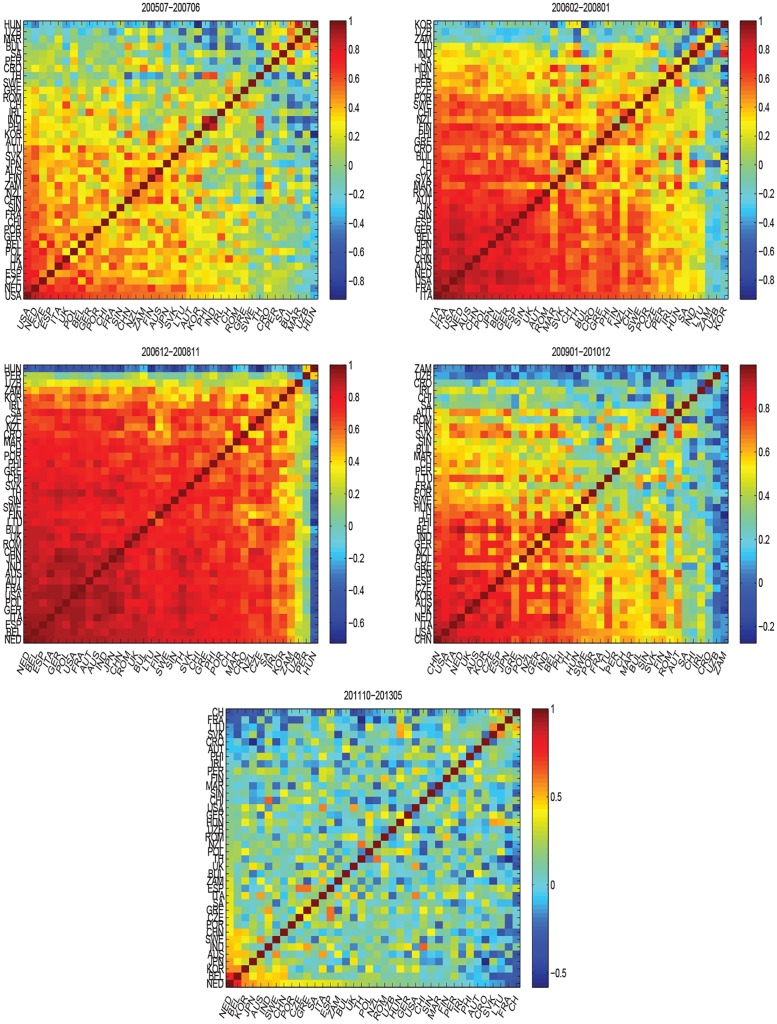
Correlation matrices *C*(*t*). The ending time *t* of the windows are 2007.06, 2008.01, 2008.11, 2010.12 and 2013.05 respectively.

To better understand the spatiotemporal dynamics of global oil market at the country level, we divide the countries into three clusters for each time *t*, as shown in Fig 8(a). Countries in the red cluster have the strongest correlations, countries in the blue cluster have the second strongest, and countries in the green cluster have the weakest. We also find, in period *P*_3_, most of countries are in red and blue, which means the risk of oil market is very high. In periods *P*_2_ and *P*_4_, more countries are in blue and green, which indicates the risk of oil market is also high. While in period *P*_5_, a majority of countries are in green, which illustrates that the correlations of oil import values of these countries weaken and the oil market risk also decreases. Fig 8(b) shows the evolution of cluster ratios. On the whole, the fraction of the giant cluster is always above 0.4. It means that most oil importers are in the same cluster, and their correlations are very close. This is consistent with Fig 8(a). Fig 9 shows correlation matrices *C*(*t*). The ending time *t* of the windows are 2007.06, 2008.01, 2008.11, 2010.12 and 2013.05 respectively. In general, the majority of counties are in the same cluster and with a weak positive correlation in June 2007 of period *P*_1_. There also exist Hungary, Uzbekistan, Morocco, Bulgaria, South Africa, Peru and Croatia having weak correlations with other countries and changing constantly. They don’t belong to the giant cluster. This indicates the world oil market is stable. In January 2008 of period *P*_2_, lots of countries are in the same cluster and with a strong positive correlation. Meanwhile there are Korea, Uzbekistan, Zambia and Lithuania, which have weak correlations with other countries. This illustrates the oil market risk exists. In November 2008 of period *P*_3_, almost all of the countries are classified into one cluster and show very strong positive correlations with each other. At the same time, very few countries like Hungary, Peru and Uzbekistan have weak correlations with other countries. This shows the oil market risk is unacceptably high. In December 2010, global oil market shows similar results with that in January 2008. Most of countries represent strong positive correlations and form a giant cluster. But countries like Zambia, Uzbekistan, Croatia and Ireland have weak correlation with others, they are not in the giant cluster. So the oil market risk remains high, but a little weaker than that in November 2008. In May 2013 of period *P*_5_, lots of countries show weak correlations, which reflect a good marketing environment. The above analysis agree well with the results of Figs 4, 6 and 8.

### 3.4 Fitness evaluation of global oil market

Further, we divide the 38 countries into clusters for each time *t* by taking consider the distance between correlation coefficients. The fraction of the giant component Φ(*t*) is defined as a ratio of the biggest cluster size to 38. Φ(*t*) reflects the overall correlation of the 38 oil importers at time *t* well. Since dependency *D*(*t*) embodies the dependency between market objects, here *D*(*t*) is the mean value of import dependency of the 38 oil importers. Absorption ratio *E*(*t*) can measure the market risk, which is the fundamental attribute of economy. This particular indicator reveals the return rate of market investment, and reflects the development level of energy infrastructure and modern service industry. Therefore *D*(*t*), *E*(*t*) and Φ(*t*) can effectively represent the operation state of the market. Here we define a market fitness evaluation function *F*(*t*) which involves *D*(*t*), *E*(*t*) and Φ(*t*). Obviously Φ(*t*) is inversely proportional to *D*(*t*), *E*(*t*) and Φ(*t*). The fitness evaluation function is expressed as the following

$$F\left(t\right)=1-(\omega_{1}D\left(t\right)+\omega_{2}E\left(t\right)+\omega_{3}\Phi \left(t\right)),$$ (11)

where the weight *ω*_*i*_(*i* = 1, 2, 3) ≥ 0, and satisfying *ω*_1_ + *ω*_2_ + *ω*_3_ = 1.

**Fig 10:**
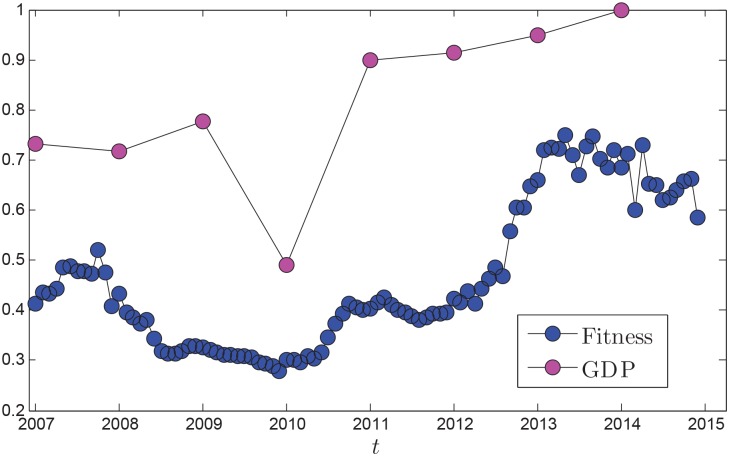
Evolutions of global oil market fitness and GDP (the totality of the 38 oil-dependent countries GDP).

By computing the correlation coefficients between GDP of oil importers (the totality of the 38 oil-dependent countries GDP) and *D*(*t*), *E*(*t*) and Φ(*t*), we get the optimal weight assignments:*ω*_1_ = 0.2219, *ω*_2_ = 0.4265, *ω*_3_ = 0.3516. Then based on Eq (11), the fitness of global oil market is evaluated. Fig 10 shows the evolution of fitness function and GDP. We find the above two evolution trends are very similar. In period *P*_2_, affected by the global financial storm, the fitness of global oil market experiences significant decreases. In period *P*_3_, the GDP growth shows a negative trend. The fitness of oil market was on a continuous decline, and a slight increase in the end. In period *P*_4_, the fitness line of oil market rises slowly and until the late, increased greatly. In period *P*_5_, the fitness line has commendable running state. Therefore, a good oil market and economic development is an undetachable unification. In other words, we can predict the fitness evolution of global oil market by the GDP predictions. This will help preventing oil supply risk by adjusting the three indices, which means that a good global oil market environment by adjusting three weight indices can be obtained.

## 4. Conclusions

In this paper, we build a directed global oil trade network with the oil-trading volume as the weight of the edge. We apply network analysis on overall topological structure properties, such as degree, strength, cumulative distribution, information entropy and weighted clustering. The structural evolution of global oil trade network during 2001–2013 is investigated as well. The result shows that the global oil import and export networks do not display typical scale-free distributions, but some local smaller countries still prefer to trade with regional powers. Because of the disassortative property, if the risk of regional major oil exporters increases, it will possibly bring great risk into the whole oil trade network, and break the stability of global oil market.

By applying random matrix theory method, we investigate the complex spatiotemporal dynamic of the global oil market from the country level by a demand-side analysis. We identify abundant information about the global oil market by deviating eigenvalues, which allows us to analyze in detail the spatiotemporal dynamics of the oil market. Additionally, five time periods are found, and the formation reasons are analyzed. The period shift points are verified by the evolution of country clusters, which also reveal the correlation of oil importers and reflect the oil market operations in each time period. We find high correlation of oil import values is corresponding to high oil market risk. To hedge this risk, we suggest that reducing dependence on a certain country and importing from more nations can help to decrease oil supply risk. Development of new sources of energy can help reduce the dependence on oil-producing countries. An international organization should be established to coordinate the relations among oil importers, and between oil importers and exporters, to pursue continuous development strategy of today and tomorrow. And an open, equal and fair system of competition rules should also be erected and maintained to help to avoid vicious competition among oil importers.

Based on the above, we construct a fitness function to assess whether global oil market works properly, and study its evolution. The result indicates that the changing trend of fitness agrees with the GDP evolution of the major oil importers. This implies the fitness of oil market needs certain guarantee from economic development. In addition, we can predict the global oil market evolution through the future GDP values of major oil importers. The research results provide useful insights and valuable reference for defending oil supply risk and formulating oil security strategy.
